# Embryonic mesenchyme, mesenchymal tumors and mesenchymal stem cells: need for clarification of cell types and standardization of biomedical terminology

**DOI:** 10.1007/s00018-025-05957-6

**Published:** 2025-11-14

**Authors:** Jörg Wilting, Jürgen Becker, Nikoloz Tsikolia

**Affiliations:** Institute of Anatomy and Cell Biology, University Medical Center Göttingen, Kreuzbergring 36, Göttingen, D-37075 Germany

**Keywords:** Biomedical terminology, Germ layers, Gastrulation, Mesoderm, Connective tissue, Stroma, EMT, MET

## Abstract

In vertebrate embryos, cellular compartments are formed with epithelial and mesenchymal arrangement of cells. During differentiation, cells can switch between these compartments several times (epithelial-mesenchymal transition; mesenchymal-epithelial transition). All germ layers produce epithelial and mesenchymal cells, so that the mesenchyme contains precursors for a large number of different cell types. Very unfortunately, practically all textbooks on anatomy and histology, and all online databases, refer to the early mesenchyme as ‘embryonic connective tissue’. Also, there is constant confusion with the term mesoderm. This has obviously influenced the definition of the terms ‘mesenchymal tumors’ and ‘mesenchymal stem cells’ in such a way that the original meaning of mesenchyme is no longer recognizable. The detection of a *bona fide* mesenchymal stem cell in adult tissue does not appear to have been successful to date, and is not to be expected, as such a stem cell does not exist in the embryo. With the aim of more precise oncological diagnostics and therapy, the heterogeneity of the cells of the connective tissue and stromal compartments in particular must be better characterized. The identification of sustentacular cells, innate lymphoid cells and immunosuppressive stromal cells is an important step in this direction. Here we discuss the term mesenchyme and argue for a more precise characterization of cell types and standardization of terminology of cells in mesenchymal tissue assemblies.

## Introduction

Even the earliest drawings of human embryos showed that there are tissues in which the cells lie close together and produce epithelial structures, as well as tissues in which the cells lie at more or less large distances from each other with plenty of intercellular space [1]. This cellular form of organization is called mesenchyme, a term introduced by the Hertwig brothers [2]. They noted that: ‘Two different forms of tissue, the epithelium and the mesenchyme, are involved in the elemental composition of the animal body,.In the epithelium, the individual cells are directly and firmly attached to each other and connected to form regular layers; in the mesenchyme, on the other hand, the connection and regular arrangement is suspended’. However, the organizational form of embryonic tissue does not give any indication of the differentiation potential of the cells. In fact, all embryonic germ layers form epithelial and mesenchymal formations, and regular transitions between the two occur, called epithelial-mesenchymal transition (EMT) and mesenchymal-epithelial transition (MET). Some cells perform several rounds of EMT and MET before final differentiation (Table 1). For example, the precursor of an angioblast that gives rise to endothelial cells of blood vessels starts his journey in the epiblast (epithelium), delaminates through the primitive streak into the early mesoderm (mesenchyme), can integrate into an early somite (epithelium), delaminates into the sclerotome or dermis (mesenchyme), and finally forms the endothelial vascular lining with a distinct luminal vs. abluminal differentiation (epithelial) [3].

**Table 1:** EMT and MET during development of selected cell types

Cell type	Blood vascular endothelial cell of body wall (Angioblast)	Striated muscle of arm and leg (Myoblast)	Melanocyte of body wall (Melanoblast)
Start of development	Epiblast (epithelial)	Epiblast (epithelial)	Epiblast (epithelial)
Developmental steps	-Early mesoderm (mesenchymal)	-Early mesoderm (mesenchymal)	-Neural plate and neural tube (epithelial)
	-Wall of early somite (epithelial)		-Neural crest and long-distance migration (mesenchymal)
	-Somite derivatives like sclerotome and dermis (mesenchymal)	-Wall of early somite (epithelial)	-Invasion into epidermis
		-Somite derivative/myotome (epithelial)	
		-Migration from myotome into limb bud (mesenchymal)	
		-Aggregation into pre-muscular mass and myotubes (epithelial-like)	
Differentiation	Vascular lining/Endothelial cells (epithelial)	Muscle fibers and satellite cells (syncytial and epithelial-like)	Melanocytes in epidermis (epithelial)

Unfortunately, almost all textbooks on anatomy and histology, and all online databases, refer to the early mesenchyme as ‘embryonic connective tissue’. This is not only imprecise and misleading, but has also done a disservice to the understanding of embryonic development and differentiation processes. For example, neural crest cells intermingle with mesoderm-derived mesenchymal cells and can only be differentiated with sophisticated methods [4]. Depending on the location in the embryo, mesenchyme contains a large number of variable progenitor cells, including (but *not* restricted to) progenitors of connective, skeletal and muscle tissues.

The term mesenchyme is also used in oncology where mesenchymal tumors are believed to origin from mesenchymal cells and embryonic mesoderm [5] and are frequently used synonymously with soft tissue and bone cancer [6, 7]. The Pschyrembel (2023) defines the term ‘mesenchymal tumor’ as: ‘Neoplasia that originates from the mesenchyme (connective, supporting or muscle tissue). Hematogenous neoplasms are an exception and form a separate group, even if blood cells originate from the mesenchyme. Mesenchymal tumors are classified according to tissue of origin and dignity’ [8]. This definition immediately makes it clear that the embryonic term mesenchyme is primarily associated with connective and skeletal tissues, but also with muscle tissue, whereby no differentiation is made between the diverse muscle types. The reference to hematogenous neoplasia also shows that there is great confusion between the terms mesenchyme and mesoderm. Hematopoietic cells originate from extra- and intra-embryonic mesoderm. They form blood-islands and intra-aortic (intraluminal) cell clusters [9–13]. The intra-aortic cell clusters, which form the life-long active hematopoietic cells, are derived from hemogenic endothelial cells. These cells can hardly be referred to as mesenchymal. Although the definitive hematopoietic cells in bone marrow might be of heterogenic origin [14], one has to ask how useful a definition is for which exceptions are immediately formulated. Confusion regarding the terms mesoderm and mesenchyme also arose from the fact that the term “mesenchymal stem cell” was introduced with reference to the developmental potential of certain mesodermal cells (chick embryo limb bud cells) [15], without taking into account the full potential of the mesoderm. In addition to cardiomyocytes, hematopoietic cells, and primordial germ cells, multiple cell types originate from the mesoderm (Table 2).

**Table 2:** Mesoderm-derived cell types (besides primordial germ cells)

Mesodermal compartment	Embryonic structure	Cell type/Organ
Axial	Notochord Prechordal mesoderm	Chorda cells, Nucleus pulposus Myoblasts (external eye muscles)
Paraxial	Preotic mesoderm Postotic mesoderm Segmental plate/Somites Tail bud	Meningeal cells All types of connective (stromal) and supporting tissue cells Vascular and lymphatic endothelial cells Smooth muscle cells Pericytes Dermal and hypodermal cells Skeletal muscle Innate lymphoid cells (?)
Intermediate	Somite stalk	Angioblasts Wolffian duct derivatives (Pro-, Mesonephros) Metanephrogenic mesenchyme (nephrons, podocytes, Bowman’s capsule)
Lateral plate (somatic and splanchnic)	Mesodermal portion of somatopleura and splanchnopleura	Cardiomyocytes All types of connective (stromal) and supporting tissue cells of body wall, extremities and internal organs Derivatives of hemangioblast Lymphatic endothelial cell Smooth muscle cells Dermal and hypodermal cells Mucosal (stromal) cells Innate lymphoid cells (?) Immunosuppressive stromal cells (?)
Coelomic epithelium (mesothelium)	Lining of coelomic cavities	Müllerian duct derivatives (inner lining of female tract) Gonadal somatic cell types Adrenal cortex endocrine cells Epicardial, pleural, and peritoneal lining Visceral vascular smooth muscle cells Pericytes

The table does not claim to be exhaustive.?=embryonic development not investigated in depth

Embryonic mesenchyme contains heterogenous progenitor cells, and there has been hope to find similar cells in the post-embryonic or adult human organism, for regenerative purposes. Theoretically, a mesenchymal stem cell (MSC) should combine all the potencies of embryonic mesenchyme. The term, however, was introduced mainly to describe progenitor cells for repair of cartilage and bone, as well as tendons, ligaments, bone marrow stroma, adipocytes, dermis, muscle, and connective tissue [15, 16]. It was later proposed to call such a cell a multipotent mesenchymal stromal cell, but the idea that a mesenchymal stem cell could exist in the adult was kept alive [17]. Here, we discuss the term mesenchyme in its various biomedical forms.

## Embryonic mesenchyme

Like in other amniotes, the human embryonic blastodisc transiently consists of an epiblast and a hypoblast. The hypoblast almost exclusively forms extra-embryonic nutritive tissues, but it is essential for the induction of gastrulation in the epiblast via the primitive streak [18–21]. The epiblast gives rise to three germ layers (ectoderm, mesoderm, endoderm) thus contributing to all embryonic cell types. During the ongoing development and differentiation process, the cells undergo morphological changes that are characterized as epithelial or mesenchymal. All three germ layers produce epithelial and mesenchymal cells. There are, however, specific regions at the cranial and caudal ends of the embryo that are not well described by these two terms. The cranial end of the early embryo is characterized by the fact that surface ectoderm, neural ectoderm, endoderm, and prechordal mesoderm are in direct contact, not separated by a continuous basement membrane [22]. Exchange of cells may take place in any direction. Additionally, in the tail bud both the ectodermal neural tube (secondary neurulation) and mesodermal compartments develop from a common neuromesodermal ‘condensation’ [23].

### Mesoderm

**Fig. 1:**
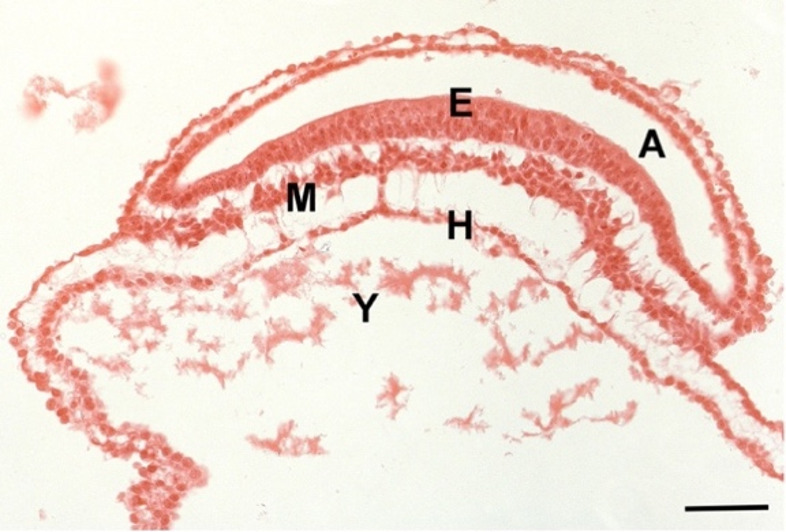
Human Embryo, Carnegie stage 8, showing epiblast (**E**), hypoblast (**H**), mesoderm (**M**), amniotic cavity (**A**), and yolk sac (**Y**). Mesodermal cells show variable intercellular space and possess long processes characteristic of mesenchymal cells. Bar = 80 μm

**Fig. 2:**
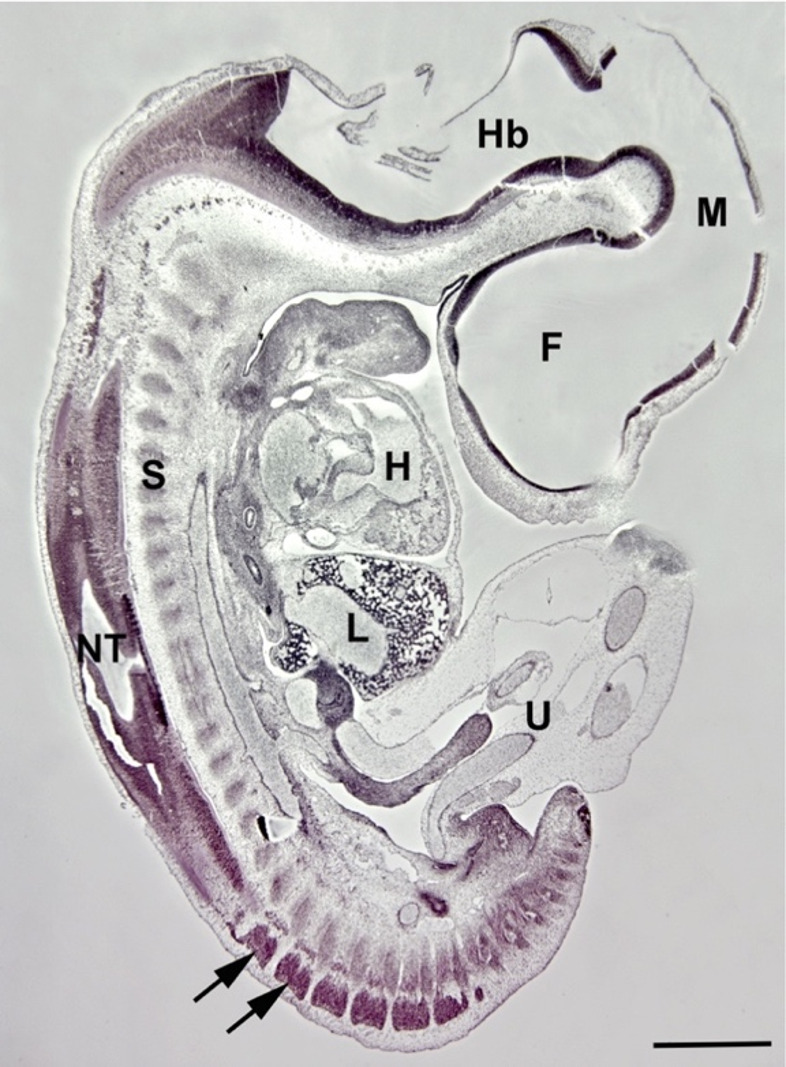
Human Embryo, 11 mm CRL, Carnegie stage 16. The development of the three germ layers is clearly advanced and organogenesis is visible. Arrows show spinal ganglia. Forebrain (**F**), heart (**H**), hindbrain (**Hb**), liver (**L**), midbrain (**M**), neural tube (**NT**), somite derivatives (**S**), umbilicus (**U**). Bar = 1 mm

The early mesoderm, which develops between the epi- and the hypoblast during gastrulation by means of ingression is, with the exception of the axial notochordal mesoderm [24], mesenchymal in its organization (Fig. 1). Through proliferation, ingression, delamination and migration the primitive streak and primitive node produce notochord, prechordal mesoderm, paraxial mesoderm, cardiac mesoderm, intermediate mesoderm, lateral plate mesoderm, primordial germ cells, extra-embryonic mesoderm and the tail bud [25–27]. As already described above for endothelial cells (Table 1), mesodermal cells can undergo EMT and MET several times during differentiation. This results in a considerable heterogeneity of cells: including connective tissue and skeletal tissues, organ-specific stromal cells, meninges, all types of muscle cells, blood vascular and lymphatic endothelial cells, hematopoietic cells, mesothelial cells, adrenal cortex, kidney, germ cells, and derivatives of the Wolffian and Müllerian ducts (Fig. 2; Table 2). Again: epithelial and mesenchymal phases can follow one another several times. For example, a myoblast that forms the skeletal muscle of the limbs starts in the epiblast (epithelium), delaminates through the primitive streak into the early mesoderm (mesenchyme), integrates into a somite and its myotome (epithelium), delaminates and migrates into the limb bud (mesenchyme), forms a pre-muscular mass and finally a syncytium with cells of the same kind (epithelial-like with basement membrane) [28].

## Ectoderm

The early embryonic ectoderm mainly consists of the anlage of the epidermis, neural tube, and ectodermal placodes (Fig. 2). The best-known example of the formation of mesenchymal cells from ectoderm (also called mesectoderm) is the neural tube-derived neural crest [29–31], which has been designated a fourth germ layer as it is a major contributor to head formation in vertebrates [32]. In the head, the neural crest produces connective, skeletal and dental tissues, as well as smooth muscle cells (which also develop from trunk neural crest and various mesodermal sources), and pericytes [11, 33] (Table 3). The trunk neural crest gives rise to peripheral nervous system (neurons, glia, sensory organs, ensheathing cells, Merkel cells), adrenal medulla and paraganglia (chromaffin cells, sustentacular cells, small intensively fluorescent cells), calcitonin-secreting cells in thyroid, and melanocytes (Table 1) [31, 34, 35]. EMT also takes place in the three epibranchial placodes which contribute sensory neurons to the ganglia of the cranial nerves VII, IX, and X [36].

**Table 3:** Neural crest-derived tissues and cell types

Cranial neural crest	Connective tissue, stromal cells, meningeal cells Innate lymphoid cells (?) Skeletal tissues Odontoblasts and cementoblasts Cells of aortico-pulmonary septum Smooth muscle cells Pericytes Neurons and glia of sensitive and parasympathetic ganglia Intramural plexus of gut Cells of aortic and carotic paraganglia Cells of dermis and hypodermis Melanocytes Corneal stroma and endothelium; cells of uvea and fibrous tunic Parafollicular C-cells of thyroid
Trunk neural crest (cardiac, vagal, sympathetic, adrenal, sacral)	Smooth muscle cells (ascending aorta, pulmonary trunk) Melanocytes Neurons and glia of sensitive and vegetative ganglia, and sensory organs (Schwann cells, capsule/satellite cells, ensheathing cells, Merkel cells) Adrenal medulla and paraganglia (chromaffin cells, sustentacular cells, small intensively fluorescent cells) Intramural plexus of gut

The table does not claim to be exhaustive.?=embryonic development not investigated in depth

## Endoderm

The early endoderm is produced during gastrulation from the epiblast through a direct succession of EMT and MET, and the hypoblast is shifted almost completely into an extraembryonic position [37]. EMT of endoderm takes place during development of the thymus, an organ that obviously receives cells from all germ layers. In mice, Pax1-positive endodermal cells of the 3rd visceral pouch can be traced into the adult where they end up as cortical thymic reticulo-epithelial cells [38]. While reticulo-epithelial cells are of endodermal and neural crest origin, the mesoderm contributes myoid cells [39], endothelial cells, and thymocytes to the organ, while Hassall’s corpuscles seem to be of ectodermal epithelial or neural crest origin [40, 41].

*In sum*,* all embryonic germ layers produce mesenchymal cells that contribute to the development of multiple or specific organs. Many cell types pass through epithelial and mesenchymal phases during their development*,* and in some cell types these phases can change several times.*

## Mesenchymal tumors

The term mesenchyme has found its way into oncology. As noted above, the term mesenchymal tumor is not always clearly defined. It mainly refers to tumors originating from connective, supporting and muscle tissue. There is a strong overlap between mesenchymal tumors and sarcomas, which can be of skeletal or soft tissue origin, and it is generally accepted that ‚mesenchymal tumors represent one of the most challenging fields of diagnostic pathology, and refinement of classification schemes plays a key role in improving the quality of pathologic diagnosis and, as a consequence, of therapeutic options’ [42, 43].

A major source of mesenchymal tumors is the connective tissue, which is probably the most poorly characterized tissue in our body. This is likely due to the fact that connective tissue varies by organ and body region; it’s like a house number. For example: Arms and legs have exactly the same cellular composition, but look differently. All organs have an individual and characteristic morphology and vascular pattern. The shaping of organs and the body, called morphogenesis, is regulated by cells that possess positional information. These are the cells of the connective and skeletal tissues, while other cells (like endothelial cells and myoblasts) are ‘naïve’ and follow the morphogenetic instructions they receive from the connective tissue [44, 45]. Thereby, *HOX* genes are major regulators of spatial identity [46], and their involvement in carcinogenesis has been increasingly recognized [47]. Each local connective and skeletal tissue possess an individual pattern of *HOX* genes. The differentiation of multiple cell types in the absence of morphogenesis can be observed in teratomas [48].

Loose connective tissue is also the primary location of macrophages (histiocytes, which can transform into fibrohistiocytic sarcomas). The classification of macrophages into M1 and M2 polarized subtypes is an important step, but certainly not the end of the typing process [49, 50]. Some dendritic cell types were originally described as macrophages, but they appear to be a separate, heterogenous group of cells [51]. Morphogenesis and function of lymphoid organs is controlled by cells that have been designated innate lymphoid cells (ILCs). They (mostly) represent tissue-resident cells and instruct local stromal cells [52, 53]. They start their function during embryonic development as lymphoid tissue-inducer cells [54]. ILCs are the innate counterpart of the adaptive lymphoid T cells. They are until now divided into five subgroups and regulate early inflammatory responses [55]. Additionally, stromal cells have been identified that limit immune responses, prevent damage and maintain tissue functions [56].

Sarcomas as a whole are rare tumors [57] and correct diagnosis of soft tissue (mesenchymal) sarcomas is challenging [42]. Comprehensive expertise in histology, immunohistochemistry, and, increasingly, molecular genetics is required [42]. And we would like to add: a good molecular embryological background would also be desirable. Soft tissue sarcomas can originate from adipocytes, (myo)fibroblasts, fibrohistiocytes, vascular cells (endothelial, epitheloid), pericytes, smooth muscle, skeletal muscle, gastrointestinal stroma (pacemaker cells, which are also present in lymphatic collectors), extra-skeletal chondro-osseous cells (exceedingly rare), peripheral nerve sheath, as well as uncertain cells such as small round cells of soft tissue and bone (Ewing’s sarcoma [EwS] including new subsets of EwS) [42, 43]. EwS was originally described as an endothelioma of bone [58] and was later associated with mesenchymal stem cells [59]. However, what is the exact definition of a mesenchymal stem cell?

## Mesenchymal stem cells

It has been suggested that a stem cell population that gives rise to variety of mesoderm-derived tissues is active in the adult where it contributes to tissue regeneration, as well as ectopic differentiation. In radiology, calcifications are frequently observed which on histological examination turn out to be ossifications. Typical examples are vascular ossification or heterotopic ossification following surgery [60–62]. The formation of cartilage and bone in embryos and adults has been associated with the term mesenchymal stem cell (MSC) [15]. Primarily, MSCs were defined as cells derived from bone marrow and periost, adherent in cell culture and able to form cells “of mesodermal phenotype” [15]. Subsequently, it was shown that equivalent cells can be isolated from a variety of tissue types [63, 64], while further studies suggested perivascular origin of MSCs [65]. Probably, the term MSC was chosen because embryonic mesenchyme was, and still is, described as ‘embryonic connective tissue’. As discussed above, embryonic mesenchyme is much more than just the anlage of connective and skeletal tissues. Not surprisingly, the term MSC, and the many synonymous terms, have been critically discussed [66]. Furthermore, the variable set of molecular markers indicates different origin and features of these cells [67]. Nevertheless, the hope that there might be a multipotent cell in the adult body (including MSC-like cells as a part of the microvasculature of all tissues) has received much attention over the last decades.

Numerous studies and clinical trials are performed with such heterogenous cells [68]. The National Library of Medicine lists 1622 clinical trials performed with MSCs (mostly bone marrow-derived) or their microvesicles; 74 of them reporting results (https://clinicaltrials.gov/search?intr=mesenchymal%20stem%20cell; last accessed Oct. 9, 2025). The studies are usually performed on small groups of patients and deal with a very heterogeneous spectrum of diseases such as: cystic fibrosis, polycystic kidney disease, type 1 diabetes, ischemic stroke, multiple sclerosis, chronic autoimmune urticaria, spinal cord injury, amyotrophic lateral sclerosis, lung rejection, erectile dysfunction, osteogenesis imperfecta, osteoarthritis, advanced glaucoma, heart function after LVAD, dental pulp regeneration, cleft lip and palate reconstruction, rheumatoid arthritis, and many others. The heterogeneity of the studies and the cells or cell components used therein makes it clear that different, yet not definitely characterized, entities are subsumed under MSC. Examples for such cell types can be the innate lymphoid cells (ILCs) and immunosuppressive stromal cells, which are mesenchymal cells that have been characterized in recent years [52, 56]. ILCs are mostly resident cells that have been identified in mucosal sites, bone marrow, secondary lymphoid organs, and numerous nonlymphoid organs [69, 70]. They can be partially replenished by circulating bone marrow-derived cells [52]. Functionally, ILCs can be classified into 5 subgroups: circulating natural killer (NK) cells, resident (helper, regulatory) ILC1, ILC2 and ILC3 cells, and lymphoid tissue inducer (LTi) cells [69, 71]. Their interaction with immunosuppressive stromal cells needs to be characterized in detail. Intestinal immunosuppressive stromal cells interact with interferon γ-producing CD8^+^ T cells and neutrophils, and limit scar formation [56]. The activities of these cells in the MSC population may have led to the observation that MSCs evoke a broad spectrum of anti-inflammatory and immune-modulatory effects rather than producing multiple non-skeletal cell types [68]. Our observations are in line with this. Murine bone marrow-derived MSCs transplanted together with murine endothelial progenitor cells promote angiogenesis and lymphangiogenesis, but do not differentiate into endothelial cells [72]. Parakrine and immunomodulatory effects of intravascularly infused MSCs (as in many clinical trials) may be beneficial in the treatment of inflammation-associated diseases, however, ectopic osteogenic differentiation would be extremely disadvantageous in this setting.

The questions of how MSCs are defined and whether multipotent MSCs are present in the adult organism also plays a major role in oncology, and for targeted tumor therapies. EwS was originally published as an endothelioma of bone, either of vascular or of lymphatic origin [58]. It was later described to possess molecular similarities with MSCs. Thereby, the MSC group of cells contained MSCs, undifferentiated bone marrow stromal cells, adipocytes and myocytes [59]. However, the immunohistological markers of EwS and its characteristic transcription factors are highly expressed in lymphatic endothelial cells [73]. Although EwS cells are genetically defined in detail [43, 74], further clarification of the cell(s)-of-origin could expand the range of therapies.

## Conclusions

The inconsistent use of the term mesenchyme is confusing and unsatisfactory for research, diagnostics, and therapy. Considering the embryonic mesenchyme as much more than just a connective or filling tissue will be helpful to understand the complexity of embryonic development and the heterogeneity of cell types derived from the various embryonic compartments. It is probably this misunderstanding that gave rise to the term MSC for the precursors of adult connective and supportive tissue. The simultaneous use of other terminology including: multipotential stromal cells, mesenchymal stromal cells, mesenchymal progenitor cells, osteogenic progenitor cells, and colony-forming unit fibroblastic (CFU-Fs) [68, 75] demonstrates the lack of clarity in this area. All embryonic germ layers produce mesenchymal cells, which often differentiate into epithelial cells, and not only into soft, connective or supporting tissues. Despite recent advances, the heterogeneity of the cells in the connective tissue of adults is far from being fully understood. It is the loose connective tissue (stroma) which actively interacts with cancer cells and must be characterized in greater detail. The discovery of innate lymphoid cells and immunosuppressive stromal cells is a step towards clarifying this issue. Single cell or single nuclear sequencing can help deciphering the heterogeneity of tissue components. However, a key prerequisite for these analyses is the provision of whole transcriptome sequencing data of characterized cell types. Clear terminology, understanding of embryonic developmental processes, detailed single cell sequencing, and more functional studies of stromal tissues are essential for the characterization of mesenchymal cells in health and disease.

## Data Availability

All data are included in the manuscript.
